# Design of Warped Stretch Transform

**DOI:** 10.1038/srep17148

**Published:** 2015-11-25

**Authors:** Ata Mahjoubfar, Claire Lifan Chen, Bahram Jalali

**Affiliations:** 1 Department of Electrical Engineering, University of California, Los Angeles, California 90095, USA; 2 California NanoSystems Institute, Los Angeles, California 90095, USA; 3 Department of Bioengineering, University of California, Los Angeles, California 90095, USA

## Abstract

Time stretch dispersive Fourier transform enables real-time spectroscopy at the repetition rate of million scans per second. High-speed real-time instruments ranging from analog-to-digital converters to cameras and single-shot rare-phenomena capture equipment with record performance have been empowered by it. Its warped stretch variant, realized with nonlinear group delay dispersion, offers variable-rate spectral domain sampling, as well as the ability to engineer the time-bandwidth product of the signal’s envelope to match that of the data acquisition systems. To be able to reconstruct the signal with low loss, the spectrotemporal distribution of the signal spectrum needs to be sparse. Here, for the first time, we show how to design the kernel of the transform and specifically, the nonlinear group delay profile dictated by the signal sparsity. Such a kernel leads to smart stretching with nonuniform spectral resolution, having direct utility in improvement of data acquisition rate, real-time data compression, and enhancement of ultrafast data capture accuracy. We also discuss the application of warped stretch transform in spectrotemporal analysis of continuous-time signals.

Time stretch dispersive Fourier transform^1^,^2^,^3^ addresses the analog-to-digital converter (ADC) bottleneck in real-time acquisition of ultrafast signals. It leads to fast real-time spectral measurements of wideband signals by mapping the signal into a waveform that is slow enough to be digitized in real-time. Combined with temporal or spatial encoding, time stretch dispersive Fourier transform has been used to create instruments that capture extremely fast optical phenomena at high throughput. By doing so, it has led to the discovery of optical rogue waves^4^, the creation of a new imaging modality known as the time stretch camera^5^, which has enabled detection of cancer cells in blood with record sensitivity^6^,^7^,^8^, a portfolio of other fast real-time instruments such as an ultrafast vibrometer^9^,^10^, and world record performance in analog-to-digital conversion^11^,^12^. The key feature that enables fast real-time measurements is not the Fourier transform, but rather the time stretch. For example, direct frequency-to-time mapping can be replaced by phase retrieval^13^ or coherent detection after the dispersion^14^ followed by back propagation.

Using warped group delay dispersion as a photonic hardware accelerator^15^, an optical signal’s intensity envelope can be engineered to match the specifications of the data acquisition back-end^16^,^17^,^18^. One can slow down an ultra-fast burst of data, and at the same time, achieve data compression by exploiting sparsity in the original data^19^. Also called anamorphic stretch transform^16^,^17^, the warped stretch transform performs a nonuniform frequency-to-time mapping followed by a uniform sampler. The combined effect of the transform is that the signal’s Fourier spectrum is sampled at a nonuniform rate and resolution. By designing the group delay profile according to the sparsity in the spectrum of the input signal, more samples are allocated to the information-rich portions of the spectrum and fewer to the information-sparse regions where they would be redundant. The only prior information needed is the sparsity of the signal’s spectral features, i.e. information about the ensemble of the signal spectrum. No instantaneous feature detection is required as long as the signal’s spectral sparsity is within the design range. As a primary application, the utility of this method has been recently demonstrated in real-time optical image compression^19^.

In conventional time stretch dispersive Fourier transform, a linear group delay profile is used to impose a non-zero constant group delay dispersion over the full bandwidth of the optical signal as shown in Fig. 1 (orange color). This profile as the kernel of the transform generates a linearly increasing frequency-dependent temporal shift across the bandwidth, which maps the optical spectrum into a temporal waveform detectable with a single-pixel photodetector. In other words, temporal dispersion stretches the optical signal in time into its Fourier transform. If the optical signal is a train of ultrafast pulses such as the output of a mode-locked laser, the spectrum of each individual pulse is mapped into a temporal waveform filling the gaps between pulses. To analyze the signal in digital domain, an analog-to-digital converter (ADC) samples the output of the photodetector at a constant rate, which can be interpreted as a uniform Fourier domain sampling of the signal spectrum. It is important to note that the value of each sample of the temporal waveform corresponds to the integral of the optical spectrum over a spectral resolvable window. For a linear group delay profile, the width of this spectral window is fixed, and it does not change over the bandwidth (see orange stripes in Fig. 1).

A warped time stretch dispersive Fourier transform is achieved by a nonlinear group delay profile as the kernel of the transform, which imposes a frequency-dependent group delay dispersion onto the spectral components. An example of a nonlinear group delay profile is shown in blue plots of Fig. 1. Here, the optical spectrum is mapped nonuniformly to time, stretching parts of the spectrum more than the other. In the example shown, the dispersion (slope of the group delay profile) in the central and peripheral parts of the spectrum is smaller than the linear profile, leading to a stretch which is less than the linear profile. However, the profile has also two regions of high dispersion, which map the spectrum into a longer and more-detailed temporal waveform compared to the linear case. It is a remarkable fact that uniform sampling of the warped time stretch output signal corresponds to nonuniform sampling of the spectrum with a frequency-dependent spectral resolvable window (see blue stripes in Fig. 1).

## Results

The warped time stretch dispersive Fourier transformation can be contemplated as a spectrotemporal operation, where its effectiveness in capturing spectral details is dictated by the sparsity and the redundancy of the input signal spectrum. Namely, sparsity in the spectrum is the attribute that influences and guides the design of the group delay dispersion profile. Note that the traditional notion of sparsity, i.e. sparsity in time or spectrum, is not pertinent here. Instead, sparsity here refers to the absence of the spectral features, i.e. abrupt variations of spectrum magnitude. The spectrum only needs to be feature-sparse; it does not need to be narrowband or contain limited number of spectral components.

**Figure 2:**
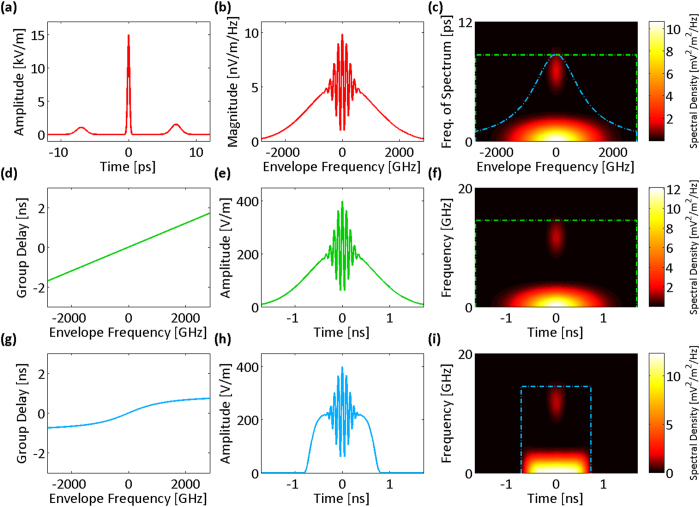
Group delay design based on spectrotemporal sparsity at the spectrum peripheries. (**a**) Envelope of the electric field of an input optical signal. (**b**) The spectrum magnitude of the input envelope. (**c**) Spectrogram of the spectrum magnitude formed by short-term Fourier transform. Here, the window of the short-term Fourier transform is slid over the envelope frequencies for the input envelope spectrum magnitude, and the Fourier transform of the signal in the window gives the local frequencies of spectrum. At the center of the spectrum magnitude, there are fast oscillations (see Fig. 2b), which result in high frequencies of spectrum. (**d**) If a temporally dispersive element with a linear group delay profile over the optical bandwidth is used to stretch the input optical field, (**e**) the spectrum maps uniformly to temporal envelope of the electric field. (**f**) Spectrogram of the envelope waveform amplitude resembles that of the spectrum magnitude (shown in Fig. 2c). The electronic acquisition time and bandwidth are marked with the green dot-dash box. Also, equivalent limitations on the envelope optical frequency and the frequency of spectrum are marked with a similar green dot-dash box on Fig. 2c. (**g**) If a nonlinear group delay profile with lower dispersion (slope of the group delay profile) at the sides of the bandwidth is used to stretch the optical pulse, (**h**) the spectrum is nonlinearly mapped to the electric field envelope in time with the sides of the spectrum stretched less than the central part. This is desirable as the sides of the spectrum magnitude do not have fast oscillations, and to capture them with a limited acquisition bandwidth, there is no need to stretch them as much as the central part. Essentially, warped stretch avoids overstretching of the spectrum peripheries unlike the linear profile (see Fig. 2e). (**i**) The spectrogram of the electric field envelope amplitude after the nonuniform dispersion shows that the power in the spectrum peripheries is squeezed toward the center, and a shorter temporal window is required to capture the waveform with the same acquisition bandwidth (see the spectrotemporal electronic acquisition window marked by the blue dot-dash box).

We describe the spectrotemporal operation of the warped time stretch with a set of examples. Without loss of generality, we initially assume that any chirp in the input signal is negligible compared to the applied group delay dispersion chirp; for example, the total temporal duration of each pulse is much shorter than the overall group delay of the dispersion profile over the pulse bandwidth. In Fig. 2a, the envelope of an optical field as the input signal of the stretch transform is shown. The spectrum of this envelope (Fig. 2b) has fast variations in the central region (feature-dense) and is relatively smooth in the wings (feature-sparse). The spectrum of the envelope can also be viewed as the spectrum of the input optical field downshifted to baseband. To examine the local properties of the spectrum, we use the short-term Fourier transform of the spectrum and form the spectrogram of the spectrum. This is equivalent to viewing the spectrum as a temporal waveform and plotting its short-time Fourier transform (Fig. 2c). As a result, the horizontal axis is the input frequency and the vertical axis is the local frequency of the variations in spectrum magnitude. We call this the frequency of spectrum, which corresponds to the period of variations in the spectrum magnitude or the temporal distance of frequency components of the signal. Hence, the local frequency bandwidth is broad in the central region and narrow in the wings (Fig. 2c). A linear group delay profile (Fig. 2d) performs conventional time stretch dispersive Fourier transform, in which the spectrum is uniformly mapped into a temporal waveform (Fig. 2e). The short-time Fourier transform of this temporal waveform (Fig. 2f) resembles that of the spectrum (Fig. 2c). To reduce the required acquisition bandwidth or time duration (memory), the spectrotemporal distribution of the signal can be reshaped by a nonlinear group delay profile as a filter, whose characteristics conform to the local frequency patterns. In the regions where the spectrum magnitude has fast variations, the filter should present a high group delay dispersion (slope) resulting in larger stretching in time than the slow varying regions of the spectrum magnitude. For the signal spectrum shown in Fig. 2b, a desired group delay profile is shown in Fig. 2g. The frequency-to-time mapping and temporal stretching are warped in such a manner that the sparse wings of the spectrum are squeezed relative to the dense central region (Fig. 2h). The sparse wings are squeezed in so that they occupy a shorter time duration after the frequency to time mapping. As a result, the reshaped spectrotemporal distribution (Fig. 2i) has the same bandwidth as the linear case, but a compressed time duration (compare dot-dashed boxes of Figs. 2f-2i). We will show that the blue dot-dashed box in Fig. 2i, which depicts the acquisition time-bandwidth limit, translates into a frequency-dependent effective bandwidth on the frequency of spectrum as shown with the blue dot-dash contour in Fig. 2c, and the spectrotemporal feature sparsity of the signal can be used to reduce the envelope acquisition time-bandwidth product in time stretch dispersive Fourier transform.

Upon uniform temporal sampling, the nonuniform mapping of warped stretch causes the information dense portion of the spectrum to effectively receive higher sampling resolution than the information sparse regions, leading to nonuniform spectral sampling. The local spectral sampling rate is basically designed to match the signal’s spectrum sparsity. We note that this nonuniform sampling is performed not by a hard-to-reach variable rate sampler, but with a uniform sampler preceded by warped spectrotemporal reshaping. This approach offers similar functionality as compressive sensing^20^,^21^ albeit it achieves it via an entirely different approach, namely that of warped time stretch dispersive Fourier transform. If the input signal is significantly chirped, the group delay profile should correspond to the difference between the desired profile for the transform limited version of the input signal (inverse Fourier transform of the input signal spectrum modulus) and the chirp of the input signal.

### Spectral Resolution

The resolution of the nonuniform spectral sampling is determined by the sampling resolution of the temporal waveform and the spectral resolution of dispersive Fourier transform. The temporal resolution of the samples is itself limited by the photodetector electrical bandwidth and the Nyquist bandwidth of the analog-to-digital converter. If we assume a resistor-capacitor circuit model for the photodetector output and use its 10% to 90% rise time as the temporal resolution^22^, the photodetector spectral resolution limit is


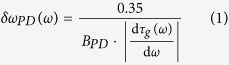


where, 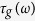 is group delay profile and *B*_*PD*_ is the electrical bandwidth of the photodetector. Also, the resolution limit in Fourier domain set by the Nyquist bandwidth of the analog-to-digital converter^23^, *B*_*ADC*_, is


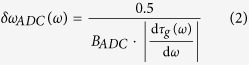


The spectral resolution of the dispersive Fourier transform imposed by ambiguity in frequency-to-time mapping is


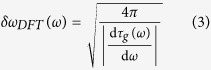


which is derived by stationary phase approximation^24^. Finally, the overall spectral sampling resolution is limited by the largest of these three at each frequency. Therefore,


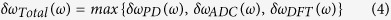


**Figure 3:**
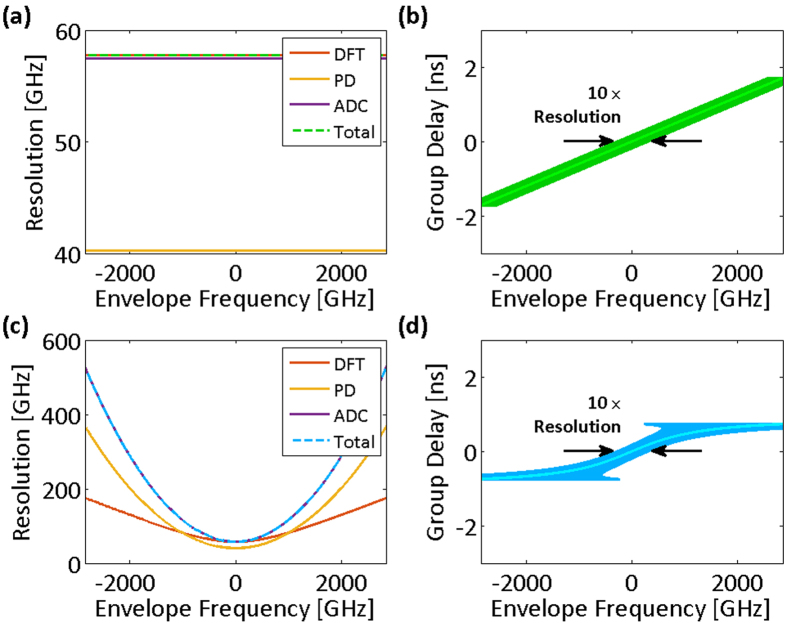
Spectral sampling resolution of time stretch dispersive Fourier transform tuned for the spectrum center. Resolution of the spectral sampling using time stretch dispersive Fourier transform is the maximum of resolution limits imposed by the temporal resolution of the photodetector, the bandwidth of the analog-to-digital converter, and the ambiguity in the frequency-to-time mapping of the dispersive Fourier transform. (**a**) Spectral resolution limits for the linear group delay profile and the acquisition system of Fig. 2d–f (photodetector and analog-to-digital converter Nyquist bandwidths are 14.5 GHz). The overall spectral sampling resolution of the linear time stretch is independent of the envelope optical frequency and limited by the ambiguity in the frequency-to-time mapping of the dispersive Fourier transform. (**b**) The spectral sampling resolution of the linear group delay profile magnified ten times (for visual clarity) and overlapped on the profile. One tenth of the overlay width at each group delay corresponds to the set of the optical frequencies that are captured at the same delay and are indistinguishable in the temporal waveform. (**c**) Spectral resolution limits for the nonlinear group delay profile and the acquisition system of Fig. 2g–i, unlike the linear stretch, depend of the envelope optical frequency (photodetector and analog-to-digital converter Nyquist bandwidths are 14.5 GHz). The overall spectral sampling resolution is limited by the ambiguity in the frequency-to-time mapping of the dispersive Fourier transform at the center of the spectrum and by the Nyquist bandwidth of the analog-to-digital converter at the peripheries of the spectrum. (**d**) Magnified spectral sampling resolution of the warped time stretch overlapped on its group delay profile clearly shows the ambiguity grows at the spectrum peripheries. DFT: dispersive Fourier transform; PD: photodetector; ADC: analog-to-digital converter; Total: overall spectral sampling resolution.

Clearly, the resolution of the nonuniform spectral sampling is frequency-dependent for a nonlinear group delay profile. Fig. 3a,c show the spectral resolutions and their limiting components for both the linear and the nonlinear group delay profiles of Fig. 2, respectively. In low group delay dispersions, temporal resolution limits, 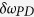 or 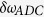, are mainly the limiting factors, whereas in high group delay dispersions, the resolution of the dispersive Fourier transform dominantly limits the spectral resolution. The spectral sampling resolution can also be translated into an effective bandwidth, *B*_*ω*_, for the frequency of spectrum. This effective bandwidth, calculated as


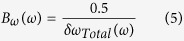


is shown with dot-dashed lines in Fig. 2c. The effective bandwidth illustrates whether the characteristics of the group delay profile match the spectrotemporal sparsity of the signal, and it proficiently guides the profile design. Fig. 3b,d show the group delay profiles of Fig. 2 overlaid with their spectral resolutions. The widths of the curves at each frequency correspond to ten times the spectral resolution. The magnification factor, ten, is used to make the subtle changes in spectral resolution more noticeable next to the group delay profile, which prominently determines it.

**Figure 4:**
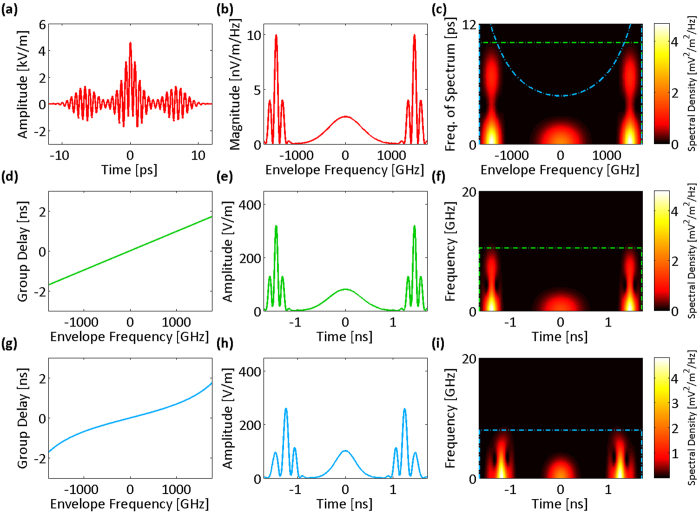
Group delay design based on spectrotemporal sparsity at the spectrum center. (**a**) Envelope of the electric field of an input optical signal. (**b**) The spectrum magnitude of the input envelope. (**c**) Spectrogram of the spectrum magnitude formed by short-term Fourier transform. At the peripheries of the spectrum magnitude, there are fast oscillations (see Fig. 4b), which result in high frequencies of spectrum. (**d**) If a temporally dispersive element with a linear group delay profile over the optical bandwidth is used to stretch the input optical field, (**e**) the spectrum maps uniformly to temporal envelope of the electric field. (**f**) Spectrogram of the envelope waveform amplitude resembles that of the spectrum magnitude (shown in Fig. 4c). The electronic acquisition time and bandwidth are marked with the green dot-dash box. Also, equivalent limitations on the envelope optical frequency and the frequency of spectrum are marked with a similar green dot-dash box on Fig. 4c. (**g**) If a nonlinear group delay profile with lower dispersion (slope of the group delay profile) at the center of the bandwidth is used to stretch the optical pulse, (**h**) the spectrum is nonuniformly mapped to the electric field envelope in time with the center of the spectrum stretched less than the peripheral parts. This is desirable as the center of the spectrum magnitude does not have fast oscillations, and to capture it with a limited acquisition time, there is no need to stretch it as much as the peripheral parts. Essentially, the warped profile avoids overstretching of the spectrum center and understretching of the spectrum peripheries, unlike linear profile (see Fig. 4e). (**i**) The spectrogram of the electric field envelope amplitude after the nonuniform dispersion shows that the power at high frequencies is squeezed toward the lower frequencies, and a smaller acquisition bandwidth is required to capture the waveform with the same temporal sampling duration (see the spectrotemporal electronic acquisition window marked by the blue dot-dash box). This time-bandwidth limit translates into a frequency-dependent effective bandwidth for the frequency of spectrum as shown with the blue dot-dash region in Fig. 4c.

**Figure 5:**
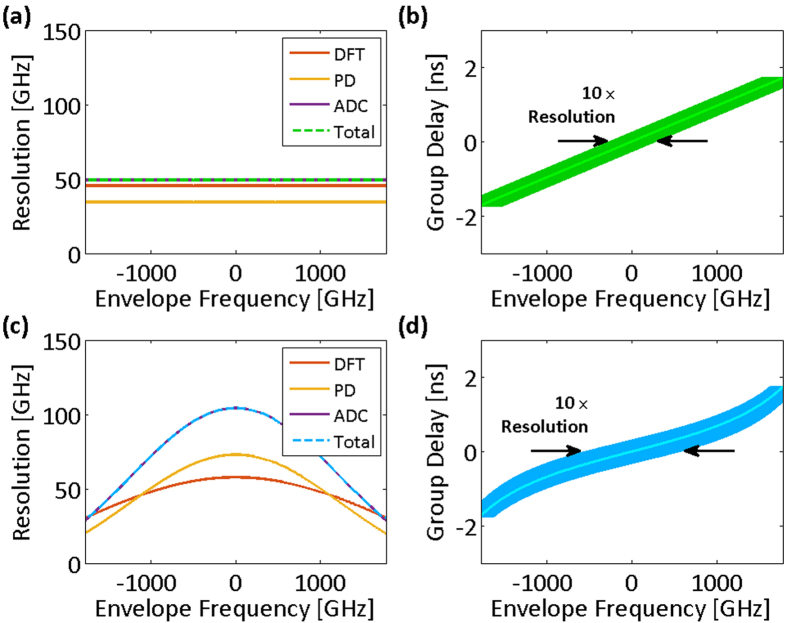
Spectral sampling resolution of time stretch dispersive Fourier transform tuned for the spectrum peripheries. (**a**) Spectral resolution limits for the linear group delay profile and the acquisition system of Fig. 4d–f (photodetector and analog-to-digital converter Nyquist bandwidths are 10.5 GHz). The overall spectral sampling resolution of the linear time stretch is independent of the envelope optical frequency and limited by the Nyquist bandwidth of the analog-to-digital converter. (**b**) The spectral sampling resolution of the linear group delay profile magnified ten times (for visual clarity) and overlapped on the profile. (**c**) Spectral resolution limits for the nonlinear group delay profile and the acquisition system of Fig. 4g–i (photodetector and analog-to-digital converter Nyquist bandwidths are 8 GHz). The overall spectral sampling resolution is limited by the ambiguity in the frequency-to-time mapping of the dispersive Fourier transform at the peripheries of the spectrum and by the Nyquist bandwidth of the analog-to-digital converter at the center of the spectrum. (**d**) Magnified spectral sampling resolution of the warped time stretch overlapped on its group delay profile clearly shows the ambiguity grows at the spectrum center. DFT: dispersive Fourier transform; PD: photodetector; ADC: analog-to-digital converter; Total: overall spectral sampling resolution.

As another example, we consider a test signal with opposite sparsity compared to that shown in Fig. 2. Figure 4 shows a signal that is sparse in the central region of the spectrum and feature-dense in the wings. The group delay profile that matches this waveform has higher dispersion, i.e., temporal stretch factor, in the wings. Here, the required acquisition bandwidth is lower for the warped group delay case compared to the linear case. Figure 5 shows the nonuniform spectral resolution corresponding to this group delay design.

### Group Delay Profile Design

The concept of effective bandwidth can be used to design an ideal group delay profile that maximally exploits the spectrotemporal sparsity of a signal. Given an acceptable signal-to-noise ratio (a tolerable spectrotemporal power loss level e.g. the noise floor of the spectrogram), the important features of the spectrogram can be contoured. At each envelope frequency, the maximum frequency of spectrum on the contour line corresponds to the desired effective bandwidth, 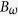, for the ideal group delay profile. It follows from Eq. (1, 2, 3), and Eq. (5) that the dispersion of the group delay profile is


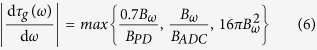


Since the ideal desired group delay profile is monotonic, it is easily derived as


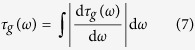


**Figure 6:**
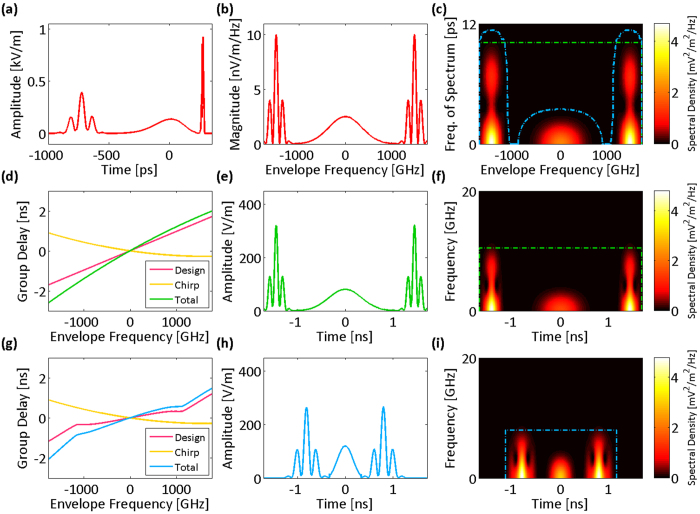
Design of an ideal group delay profile based on spectrotemporal sparsity and input signal chirp. (**a**) Envelope of the electric field of an input optical signal. (**b**) The spectrum magnitude of the input envelope. (**c**) Spectrogram of the spectrum magnitude formed by short-term Fourier transform. The region of the spectrogram with power density 30 dB less than the peak power density is contoured with a blue dot-dash line. This contour is considered as the necessary effective bandwidth to design an ideal group delay profile. (**d**) To perform uniform frequency-to-time mapping, a linear group delay design minus the input signal chirp should be used to stretch the input optical field. (**e**) The spectrum maps uniformly to temporal envelope of the electric field by the total group delay profile. (**f**) Spectrogram of the envelope waveform amplitude resembles that of the spectrum magnitude (shown in Fig. 6c). The electronic acquisition time and bandwidth are marked with a green dot-dash box. Also, equivalent limitations on the envelope optical frequency and the frequency of spectrum are marked with a similar green dot-dash box on Fig. 6c. (**g**) If a nonlinear group delay profile is designed based on the blue dot-dash contour in Fig. 6c and the input signal chirp, (**h**) the spectrum is nonlinearly mapped to the electric field envelope in time in such a way that the acquisition time is minimized for the set acquisition bandwidth (8 GHz) and the enforced spectrotemporal accuracy level (−30 dB). Essentially, the warped profile ideally avoids overstretching of the spectrum center and understretching of the spectrum peripheries, unlike the linear profile (see Fig. 6e). (**i**) The spectrogram of the electric field envelope amplitude after the nonuniform dispersion shows that the power in the spectrum peripheries is squeezed toward the center, and a shorter temporal window and acquisition bandwidth is required to capture the waveform compared to the linear case (acquisition window is marked by the blue dot-dash box). This time-bandwidth limit translates into the frequency-dependent effective bandwidth as shown with the blue dot-dash box in Fig. 6c, used for the design of the ideal group delay profile.

**Figure 7:**
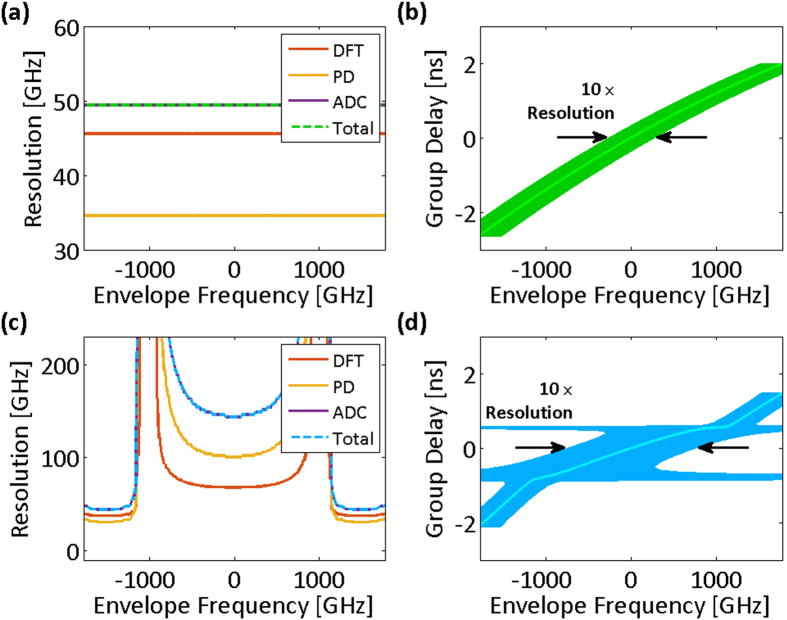
Spectral sampling resolution of time stretch dispersive Fourier transform designed for ideal exploitation of the spectrotemporal sparsity. (**a**) Spectral resolution limits for the chirp compensated linear group delay profile and the acquisition system of Fig. 6d–f (photodetector and analog-to-digital converter Nyquist bandwidths are 10.5 GHz). The overall spectral sampling resolution of the linear time stretch is independent of the envelope optical frequency and limited by the Nyquist bandwidth of the analog-to-digital converter. (**b**) The spectral sampling resolution of the linear group delay profile minus chirp magnified ten times (for visual clarity) and overlapped on the profile. One tenth of the overlay width at each group delay corresponds to the set of the optical frequencies that are captured at the same delay and are indistinguishable in the temporal waveform. (**c**) Spectral resolution limits for the nonlinear group delay profile and the acquisition system of Fig. 6g–i, unlike the linear stretch, depend of the envelope optical frequency (photodetector and analog-to-digital converter Nyquist bandwidths are 8 GHz). The overall spectral sampling resolution is limited by the Nyquist bandwidth of the analog-to-digital converter. (**d**) Magnified spectral sampling resolution of the warped time stretch overlapped on its group delay profile clearly shows the ambiguity grows at the regions of the spectrum that do not contain significant information. DFT: dispersive Fourier transform; PD: photodetector; ADC: analog-to-digital converter; Total: overall spectral sampling resolution.

The spectrogram that is used here to design the ideal group delay profile, does not display the effect of the input signal chirp because it is formed from the spectrum magnitude. If the input chirp is not negligible, it must be subtracted from the Eq. 7 to get the total group delay profile, which performs the desired frequency-to-time mapping. Figure 6a shows a chirped input signal with a spectrum magnitude (Fig. 6b) same as that of Fig. 4b. The spectrogram is contoured at −30 dB of the peak power density (blue dot-dash line in Fig. 6c), which specifies the required effective bandwidth of the frequency of spectrum for an ideal group delay profile. We compare using a linear design for the group delay profile (Fig. 6d) with designing the ideal group delay profile according the effective bandwidth by Eq. (7) (Fig. 6g). In either case, the chirp of the input signal is subtracted from the group delay design to cancel the effect of the input chirp. Thus, the total group delay profiles preserve the desired forms of frequency-to-time mappings (Fig. 6e,h). Clearly, the power in the sparse regions of the spectrogram is concentrated by the nonlinear profile (Fig. 6i) compared to that by the linear profile (Fig. 6f), which corresponds to a reduction in the required acquisition time and bandwidth. Figure 7 shows the spectral resolution of the linear and warped stretch transforms with the profiles in Fig. 6. The linear profile has a uniform resolution across the bandwidth, whereas the resolution of the nonlinear profile is frequency-dependent and designed to match the sparsity of the spectrotemporal distribution.

**Figure 8:**
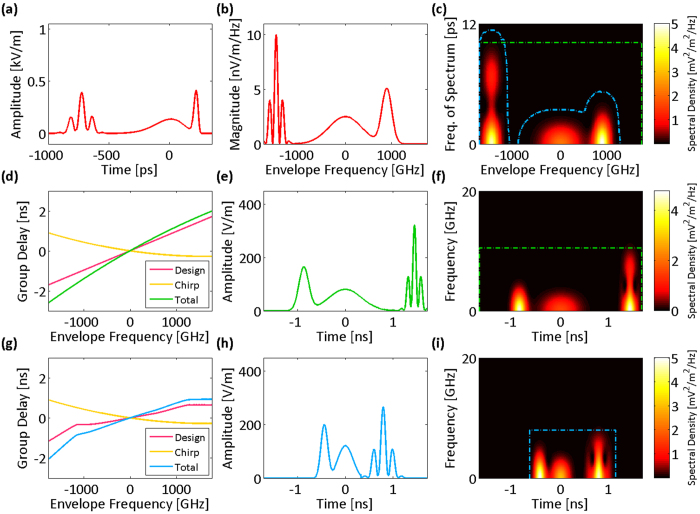
Design of an ideal group delay profile for a signal with asymmetric spectrum about the carrier frequency. (**a**) Envelope of the electric field of an input optical signal. (**b**) The spectrum magnitude of the input envelope is asymmetric. (**c**) Spectrogram of the spectrum magnitude formed by short-term Fourier transform. The region of the spectrogram with power density 30 dB less than the peak power is contoured with a blue dot-dash line. This contour is considered as the necessary effective bandwidth to design an ideal group delay profile. Note that this contour also becomes asymmetric about the carrier frequency. (**d**) To perform uniform frequency-to-time mapping, a linear group delay design minus the input signal chirp should be used to stretch the input optical field. (**e**) The spectrum maps uniformly to temporal envelope of the electric field by the chirp-corrected linear group delay profile. The frequencies higher than the center frequency experience relatively positive group delays meaning they lag behind the center frequency. (**f**) Spectrogram of the envelope waveform amplitude resembles that of the spectrum magnitude (shown in Fig. 8c). The electronic acquisition time and bandwidth are marked with a green dot-dash box. Also, equivalent limitations on the envelope optical frequency and the frequency of spectrum are marked with a similar green dot-dash box on Fig. 8c. (**g**) If a nonlinear group delay profile is designed based on the blue dot-dash contour in Fig. 8c and the input signal chirp, (**h**) the spectrum is nonuniformly mapped to the electric field envelope in time in such a way that the acquisition time is minimized for the set acquisition bandwidth (8 GHz) and the enforced spectrotemporal accuracy level (−30 dB). (**i**) The spectrogram of the electric field envelope amplitude after the nonuniform dispersion shows that a shorter temporal window and a smaller acquisition bandwidth are sufficient to capture the waveform compared to the linear case (the blue dot-dash box). This time-bandwidth acquisition window translates into the frequency-dependent effective bandwidth as shown with the blue dot-dash contour in Fig. 8c, which was used to design the nonlinear group delay profile.

**Figure 9:**
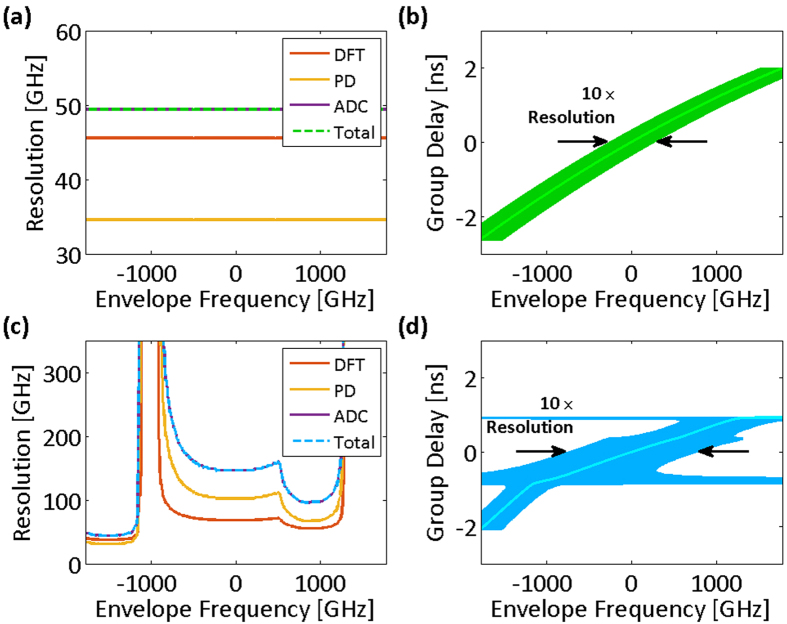
Spectral sampling resolution of time stretch dispersive Fourier transform designed according to the spectrotemporal sparsity for a signal with asymmetric spectral features. (**a**) Spectral resolution limits for the chirp compensated linear group delay profile and the acquisition system of Fig. 8d–f (photodetector and analog-to-digital converter Nyquist bandwidths are 10.5 GHz). The overall spectral sampling resolution of the linear time stretch is independent of the envelope optical frequency and limited by the Nyquist bandwidth of the analog-to-digital converter. (**b**) The spectral sampling resolution of the linear group delay profile minus chirp magnified ten times (for visual clarity) and overlapped on the profile. (**c**) Spectral resolution limits corresponding to the nonlinear group delay profile and the acquisition system of Fig. 8g–i, which unlike the linear stretch, depend of the envelope optical frequency (photodetector and analog-to-digital converter Nyquist bandwidths are 8 GHz). In this case, the overall spectral sampling resolution is limited by the Nyquist bandwidth of the analog-to-digital converter. (**d**) Magnified spectral sampling resolution of the warped time stretch overlapped on its group delay profile clearly shows the ambiguity grows at the regions of the spectrum that do not contain sharp spectral features. DFT: dispersive Fourier transform; PD: photodetector; ADC: analog-to-digital converter; Total: overall spectral sampling resolution.

To further show the applicability of our design method, we consider a signal (Fig. 8a) with asymmetric spectrum about the carrier frequency (Fig. 8b). This corresponds to a signal with complex temporal envelope (In Fig. 8a, we are showing the absolute value of the complex envelope). Using our design algorithm, the spectrogram is contoured at −30 dB of the peak power density (blue dot-dash line in Fig. 8c), specifying the required effective bandwidth of the frequency of spectrum for an ideal group delay profile. If we use a chirp-compensated linear group delay profile (Fig. 8d), the spectrum to time mapping would be uniform. Of course, this results in an asymmetric temporal waveform (Fig. 8e), which resembles the input signal spectrum (Fig. 8b). The vertical flip is due to higher frequencies receiving larger group delays, lagging more behind. The spectrogram of the temporal envelope shows the same type of flip (Fig. 8f). If we use the nonlinear group delay profile designed with our technique (Fig. 8g) to stretch the signal nonuniformly (Fig. 8h), compared to the linear case (Fig. 8f), a shorter time duration and a smaller acquisition bandwidth are sufficient (Fig. 8i). Clearly, even in the case of a signal with asymmetric spectrum, the design algorithm leads to a nonlinear group delay profile, which efficiently reduces the time-bandwidth product of the envelope by warped stretch transform. For the group delay profiles shown in Fig. 8, the spectral resolutions of the linear and warped stretch transforms are depicted in Fig. 9. The linear profile has a fixed resolution across the bandwidth (Fig. 9a,b), but the resolution of the nonlinear profile is frequency dependent (Fig. 9c,d). For example, the algorithm has designed the nonlinear profile in such a way that it allocates better resolution to the parts of the spectrum that contain fast variations (feature-dense regions).

## Discussion

The nonuniform sparse Fourier domain sampling described above may be used for data compression. This works when some frequencies carry more information than others. Such frequencies are coded with fine resolution preserving features of spectrum at these frequencies. On the other hand, less important frequencies are coded with a coarser resolution. Naturally, some of the finer details of less important frequencies will be lost in the coding.

The information of interest is usually encoded into the magnitude of the spectrum, therefore a simple unwarping of the time-to-spectrum map is sufficient for reconstruction. For a more general case where the information is contained in both the amplitude and phase, reconstruction requires either coherent detection or recovery of phase from amplitude measurements. The input signal is then recovered by simulation of back propagation through the dispersive profile (filter). Generally known as a phase retrieval method, there are numerous digital algorithms available for recovering the complex amplitude from intensity-only measurements^25^,^26^.

The reconstruction accuracy and lossy nature of this compression have been analyzed previously^18^. The system reshapes the spectrotemporal structure of the signal such that nearly all the signal energy is within the bandwidth of the photodetector and the real-time digitizer of the acquisition system. Because of the limited resolution of dispersive Fourier transform by ambiguity in frequency-to-time mapping, the limited bandwidths of the photodetector and the digitizer, and the limited resolution of the digitizer, as measured by its effective number of bits (ENOB), the reconstruction will never be ideal, and therefore, this is a lossy compression method. In general, for any time-limited pulse, the spectrum is not bandlimited, and the signal reconstruction will suffer from the loss of out-of-band spectral components in the acquisition system. If the temporal width of the input signal is small enough, so that the spectral resolution is sufficient for capturing details of the input signal spectrum (i.e. in the far field), the bandwidth limitations imposed by the acquisition system can be considered as a frequency-dependent effective bandwidth on the input signal frequency of spectrum as shown in Figs. 2c, 4c, 6c and 8c. The effective bandwidth interpretation facilitates the design of group delay profile for a set of target signals with known spectral characteristics and determines the minimum amount of loss in the compression process.

As a consequence of dispersion, temporal features are transformed and slowed down in time. The amount by which a particular temporal feature is stretched is proportional to the bandwidth of the feature and the overall dispersion over the bandwidth. Fast temporal features have larger bandwidth, and as a result, they are transformed and stretched more than slow temporal features. This feature selective stretch has been referred to as a type of self adaptivity through which the output adapts to the input even when the transfer function of the system is static^27^.

The group delay dispersion profile is designed according to the spectral sparsity of the input signal as described, i.e. smart stretching. Beyond that, the group delay is static; in other words, it does not need to be dynamically varied according to the instantaneous behavior of the signal. This is why the time stretch dispersive Fourier transform as well as its warped counterpart were called self-adaptive^27^. However, if the spectral characteristics of the signal slowly varies over time, a feedback mechanism can be used to adapt the group delay profile, and subsequently the effective bandwidth to the sparsity requirements. The sophisticated group delay profiles designed by our algorithm can be readily implemented by chirped fiber Bragg grating (CFBG) technology^16^,^19^. Another implementation option is to use chromo-modal dispersion (CMD) device^28^,^15^, which uses the large modal dispersion of multimode waveguides in conjunction with the angular dispersion of diffraction gratings to create huge chromatic dispersion. For either technology, the implemented group delay profile will have some deviations from the design. A numerical study of the tolerance to profile nonidealities is performed previously^17^.

Time stretch dispersive Fourier transform maps the spectrum of the pulses in a burst-mode signal to the silent intervals in between them. In order to use the time stretch transform for the acquisition of the spectrotemporal evolution of a continuous-time signal, the signal needs to be segmented into multiple pulse trains in a process, which is called virtual time gating^29^. The pulse trains are independently dispersed by linear or warped time stretch systems in parallel, and the acquired signals are digitally stitched together to reveal the spectral features of the continuous-time signal as it varies with time. If the temporal duration of each time gate window is very small, that is, the sliced segments of the continuous-time signal are very short, the resolution of the linear or warped dispersive Fourier transform can be limited by the bandwidth of the gating window. Also, the temporal durations of the time gate windows can be different.

**Figure 10:**
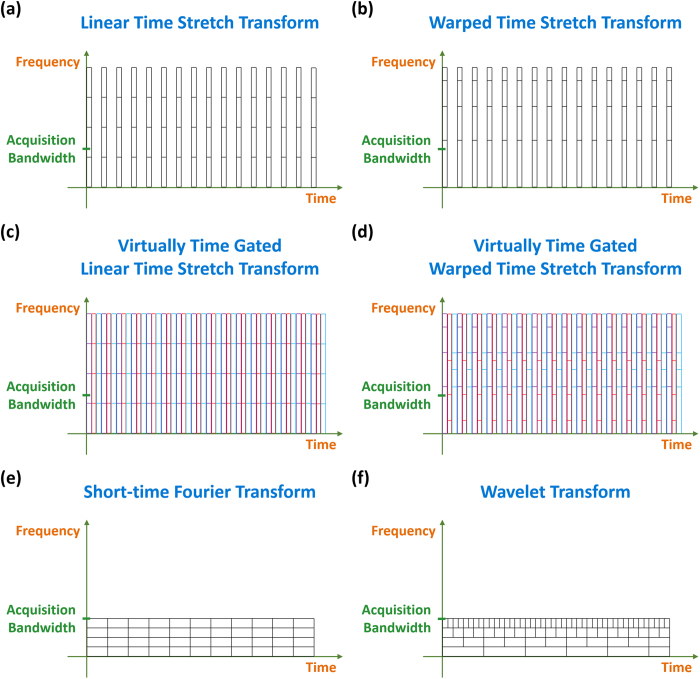
Spectrotemporal resolution of time stretch dispersive Fourier transform vs short-time Fourier transform and wavelet transform. For burst mode signals, time stretch dispersive Fourier transform takes advantage of the empty intervals between pulses to map their spectra into temporal waveforms and captures the signals with a bandwidth far beyond the acquisition bandwidth of the electronic back-end e.g. analog-to-digital converter and photodetector. (**a**) In linear time stretch, the spectral resolution is uniform, and the temporal resolution is same as the input pulse width. (**b**) In warped time stretch, the spectral resolution is nonuniform as discussed earlier, and the temporal resolution is again same as the input pulse width. (**c**) To use time stretch dispersive Fourier transform for analysis of continuous-time signals, the signal should be segmented into multiple burst mode signals. This process is called virtual time gating and can be used for both linear and warped stretch transforms. The temporal durations of the gates should not be very short to limit the spectral sampling resolution, but can be different. In virtually time gated time stretch transform, all of the gates (shown with different colors) have the same uniform spectral resolution if identical dispersions and back-end electronics are used. (**d**) In addition, for virtually time gated warped time stretch transform, the gates can have different distributions of nonuniform spectral resolutions. (**e**) Short-time Fourier transform can also be used to digitally generate the spectrotemporal distribution of an already acquired signal, but its bandwidth is limited to that of the electronic acquisition system. (**f**) The bandwidth of digitally-implemented wavelet transform is also restricted to the electronic acquisition bandwidth, but its temporal resolution can be nonuniform.

Linear time stretch dispersive Fourier transform acquires the spectrum of each pulse with uniform spectral resolution up to frequencies far beyond the electrical acquisition bandwidth of the analog-to-digital converter and photodetector (Fig. 10a). Warped time stretch dispersive Fourier transform has the same properties as its linear counterpart, but its spectral resolution is not uniform across the bandwidth (Fig. 10b). This nonuniformity can be designed to match the spectrotemporal sparsity of the signal and therefore, increase the spectral resolution at desired frequencies under the same envelope time-bandwidth product. Both linear and warped time stretch dispersive Fourier transforms can be used in conjunction with virtual time gating technique for acquisition of the continuous-time signals (Fig. 10c,d). In virtually time gated warped stretch transform, gates can have dissimilar group delay profiles corresponding to different distributions of the nonuniform spectral resolution and be suitable for various types of spectral sparsity (Fig. 10d).

Spectrotemporal characteristics of a signal can also be analyzed digitally by capturing the signal using an analog-to-digital converter and performing short-time Fourier transform or wavelet transform on the samples. However, in these cases, the maximum frequency that can be measured is limited by the electronic acquisition bandwidth (Fig. 10e,f). The wavelet transform can also generate nonuniform temporal resolution for the spectrotemporal distribution of the signal while keeping the spectral resolution fixed (Fig. 10f).

## Conclusion

Time stretch dispersive Fourier transform is an indispensable tool for acquisition and analysis of the wideband signals at frequencies far beyond the acquisition bandwidth of the electronic back-end i.e. digitizer and photodetector. The more general form of it, warped time stretch, offers nonlinear mapping of spectrum to time, which leads to a nonuniform sampling of the spectrum. We analyzed the spectral resolution of the warped time stretch dispersive Fourier transform and defined an effective bandwidth for the transform, which guides the design of a proper group delay profile based on the spectral sparsity of the signal. Finally, linear and warped time stretch transforms are compared to other methods such as wavelet transform for spectrotemporal analysis of continuous-time signals.

## Methods

### Simulation model

We used a discrete-time complex-envelope simulation model for the analysis of warped stretch transform. The carrier frequency is assumed to be 200 THz, resembling an optical wavelength of 1.5 µm. The signal spectra are downshifted from carrier frequency to baseband, easing the required temporal resolution of the simulations. The temporal and spectral resolutions at baseband simulations are set at 0.1 ps and 150.15 MHz, respectively. The output complex envelope spectrum, 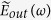, is calculated as the product of the input complex envelope spectrum, 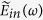, and the impulse response of the downshifted dispersion profile:


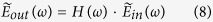


Here, *ω* is the modulation frequency, and the impulse response, *H*(*ω*), is a frequency-dependent phase shift filter;


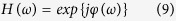


where the phase shift, 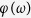, corresponds to the integral of the group delay profile; i.e.,


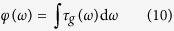


Furthermore, linear interpolation is employed to generalize designed group delay to arbitrary frequencies. If any, the chirp of the signal is derived by a moving-window short-time Fourier transform.

### Spectrograms

Spectrograms depend on the estimation method of power spectral density, e.g. the window size of the short-term Fourier transform. If the width of the short-time Fourier transform window is reduced, the time resolution of the spectrogram improves, but its frequency resolution degrades. For the frequency-of-spectrum spectrogram, these changes in window size alter the effective bandwidth contour and lead to variations in the design of the group delay profile. In other words, the width of the spectral window should be small enough to capture localized fluctuations of the spectral sparsity, but not too narrow to overestimate the effective bandwidth. Alternatives to spectrogram, for example instantaneous frequency estimated from the analytic form of the signal calculated by the Hilbert transformation, can also be used to determine the required effective bandwidth and design the group delay profile.

## Additional Information

**How to cite this article**: Mahjoubfar, A. *et al.* Design of Warped Stretch Transform. *Sci. Rep.* **5**, 17148; doi: 10.1038/srep17148 (2015).

## Figures and Tables

**Figure 1:**
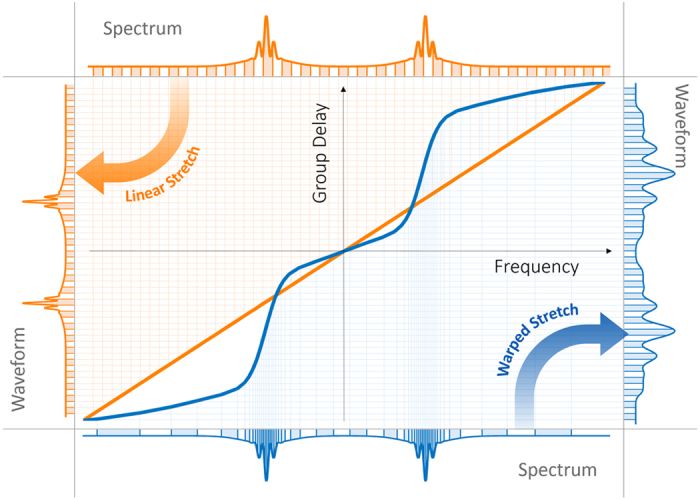
Linear and warped time stretch dispersive Fourier transforms. In linear time stretch (orange plots), a linear group delay profile with significant group delay over the signal bandwidth is used as the kernel to delay various spectral components differently, but with a constant group delay dispersion (slope of the group delay profile). If the input signal is a pulse train, pulse spectra are linearly mapped to the silent times in between the pulses. If a digitizer with constant sampling rate is used to capture the stretched pulse waveforms, the spectral sampling resolution is uniform across the bandwidth (see orange bars). In contrast, a nonlinear group delay profile (blue plots) with varying group delay dispersion over the bandwidth (different slopes) can stretch the signal spectrum nonlinearly, in which parts of the spectrum are stretched more than the others. This can be used, even with a constant rate sampler, to increase the spectral resolution at regions of the bandwidth where higher resolution is required and to reduce the resolution where the spectral features are sparse (see blue bars). If the sparsity of the spectral features, that is, regions of spectrum with fast varying magnitude or higher frequency of spectrum, is known, the nonlinear group delay profile can be designed accordingly to stretch those regions more than the linear case. Conversely, regions with low bandwidth for the frequency of spectrum can be stretched less than the linear profile. In this way, the spectrum is warp stretched into a waveform with the same temporal duration as the linear profile, but smaller bandwidth. In other words, for signals with spectral sparsity, the envelope time bandwidth product can be reduced.
